# Improvement of Alginate Extraction from Brown Seaweed (*Laminaria digitata* L.) and Valorization of Its Remaining Ethanolic Fraction

**DOI:** 10.3390/md22060280

**Published:** 2024-06-15

**Authors:** Ivana M. Savić Gajić, Ivan M. Savić, Aleksandra M. Ivanovska, Jovana D. Vunduk, Ivana S. Mihalj, Zorica B. Svirčev

**Affiliations:** 1 Faculty of Technology in Leskovac, University of Nis, Bulevar oslobodjenja 124, 16000 Leskovac, Serbia; savicivan@tf.ni.ac.rs; 2 Innovation Center of the Faculty of Technology and Metallurgy, University of Belgrade, Karnegijeva 4, 11000 Belgrade, Serbia; aivanovska@tmf.bg.ac.rs; 3 Institute of General and Physical Chemistry, Studentski Trg 12/V, 11158 Belgrade, Serbia; jvunduk@iofh.bg.ac.rs; 4 Department of Biology and Ecology, Faculty of Sciences, University of Novi Sad, Trg Dositeja Obradovica 2, 21000 Novi Sad, Serbia; ivana.mihalj@dbe.uns.ac.rs (I.S.M.); zorica.svircev@dbe.uns.ac.rs (Z.B.S.); 5 Faculty of Science and Engineering, Biochemistry, Åbo Akademi University, Tykistökatu 6A, 20520 Turku, Finland

**Keywords:** ultrasound, alginate, ethanolic fraction, chemical composition, ICP-OES, UHPLC-ESI-MS/MS, antioxidative activity, antimicrobial activity

## Abstract

This study aimed to improve the conventional procedure of alginate isolation from the brown seaweed (*Laminaria digitata* L.) biomass and investigate the possibility of further valorization of the ethanolic fraction representing the byproduct after the degreasing and depigmentation of biomass. The acid treatment of biomass supported by ultrasound was modeled and optimized regarding the alginate yield using a response surface methodology based on the Box–Behnken design. A treatment time of 30 min, a liquid-to-solid ratio of 30 mL/g, and a treatment temperature of 47 °C were proposed as optimal conditions under which the alginate yield related to the mass of dry biomass was 30.9%. The use of ultrasonic radiation significantly reduced the time required for the acid treatment of biomass by about 4 to 24 times compared to other available conventional procedures. The isolated alginate had an M/G ratio of 1.08, which indicates a greater presence of M-blocks in its structure and the possibility of forming a soft and elastic hydrogel with its use. The chemical composition of the ethanolic fraction including total antioxidant content (293 mg gallic acid equivalent/g dry weight), total flavonoid content (14.9 mg rutin equivalent/g dry weight), contents of macroelements (the highest content of sodium, 106.59 mg/g dry weight), and microelement content (the highest content of boron, 198.84 mg/g dry weight) was determined, and the identification of bioactive compounds was carried out. The results of ultra high-performance liquid chromatography–electrospray ionization–tandem mass spectrometry analysis confirmed the presence of 48 compounds, of which 41 compounds were identified as sugar alcohol, phenolic compounds, and lipids. According to the 2,2-diphenyl-1-picrylhydrazyl assay, the radical scavenging activity of the ethanolic fraction (the half-maximal inhibitory concentration of 42.84 ± 0.81 μg/mL) indicated its strong activity, which was almost the same as in the case of the positive control, synthetic antioxidant butylhydroxytoluene (the half-maximal inhibitory concentration of 36.61 ± 0.79 μg/mL). Gram-positive bacteria (*Staphylococcus aureus*, *Enterococcus faecalis*, and *Bacillus cereus*) were more sensitive to the ethanolic fraction compared to Gram-negative bacteria (*Escherichia coli*, *Pseudomonas aeruginosa*, and *Shigella sonnei*). The obtained results indicated the possibility of the further use of the ethanolic fraction as a fertilizer for plant growth in different species and antifouling agents, applicable in aquaculture.

## 1. Introduction

For almost 150 years, different species of seaweed have been exploited as alginate sources. Brown seaweed species like *Ascophyllum nodosum*, *Laminaria* sp., *Lessonia nigrescens*, *Ecklonia makima*, *Macrocystis pyrifera*, and *Durvillaea antarctica* have several advantages, which are called “environmentally friendly” [1]. However, the traditional extraction process is still far from sustainable and requires improvements starting from seaweed cultivation, the inclusion of less-exploited species in the byproduct’s treatments, and valorization [1,2]. Ultrasound-assisted extraction (UAE) [3], microwave-assisted extraction [4], and enzymatic extraction represent the most effective extraction procedures of alginate [5].

The commercial production of alginate from brown seaweed is still based on conventional acid/base procedures that are carried out in several steps (depigmentation and defatting of biomass, acid treatment, alkaline extraction, precipitation, bleaching, and drying process) [6]. Among these steps, alkaline extraction is considered the most critical factor affecting the yield and the physicochemical properties of the final product. In the literature [7], the influence of the extraction parameters on the yield and physical–chemical properties of the extracted alginate was described in detail. The extraction temperature, alkali concentration, liquid-to-solid ratio, and extraction time were the most frequently examined. Among these parameters, the extraction temperature and extraction time have a significant effect on the yield, especially at higher values. At the same time, the higher temperatures and longer extraction times can degrade alginate molecules, affecting the reduction in molecular weight (lower than 32,000–340,000 g/mol) and leading to a technologically inferior product [8]. Because of that, temperatures lower than 80 °C and extraction times of up to several hours are recommended for the alginate extraction conditions [9]. In traditional maceration procedures used for the extraction of alginate from seaweed, the acid treatment, which precedes the alkaline extraction, takes quite a long time, from 2 h [10] up to even 12 h [11]. 

The development of alginate extraction from brown seaweed follows the sustainability goals and, thus, moves toward green extraction techniques. The UAE [3], microwave-assisted extraction [4], and enzymatic extraction represent the most effective extraction procedures of alginate [5]. During the alginate production process, the rest of the biomass (about 90%) together with residual solvents is considered waste. Residual biomass can be further used to obtain minerals, proteins, and fiber cellulose [12]. Algal biomass waste has been examined and proposed as an integral part of several circular economy streamlines, including fertilizer, animal feed, food industry additives, biodegradable woven material used to treat wounds, and fiber for the clothing industry [13].

The RECAP project (funded by the Ministry of Science, Technological Development and Innovation of the Republic of Serbia) has a goal of using alginate isolated from brown seaweed (*Laminaria digitata*) biomass for the hydrogel preparation needed to impregnate the carpet necessary for cyanobacterial growth support. The main use of this carpet will be for the bioremediation of contaminated soil in the area of thermal power plants. By the way, *L. digitata* is one of the most cultivated brown seaweed species used as a source of bioactive compounds in designing new products that are significant in the human therapy of different diseases [14,15]. This is another reason for the further exploitation of *L. digitata* species. From the point of view of industrial and sustainable development, acid treatment of biomass is considered inefficient due to its longer treatment time, resulting in high labor costs. Therefore, the conventional alginate extraction procedure requires innovations to improve its efficiency. To the best of our knowledge, the acid treatment of biomass has not yet been optimized according to maximal alginate yield. Having this in mind, this inefficient process will try to be improved by including the ultrasound because it can significantly enhance alginate yield by increasing its mass transfer, allowing for better penetration of used solvent into the plant material and facilitating the release of soluble polysaccharides. In this approach, the appearance of the hydration process softens the plant material, leading to a more efficient process of alginate extraction. The obtained ethanol fraction, which is a byproduct of the defatting and depigmentation of biomass in the alginate extraction process, was subjected to further analysis of its chemical composition (total antioxidant content, total flavonoid content, identification of bioactive compounds, concentrations of macroelements and microelements), as well as antimicrobial and radical scavenging activities, to evaluate its applicative use.

## 2. Results and Discussion

The multistep industrial process of alginate isolation from brown seaweed consists of acid treatment with hydrochloric acid, which lasts a long time. Because of that, this study aimed to overcome this problem and reduce the time of biomass treatment by optimizing the ultrasound-assisted process according to the maximal alginate yield. In the industry, the ethanolic fraction that occurs in this step during the defatting and depigmentation of biomass is not valorized and represents a byproduct during alginate isolation. Because of that, the properties and chemical composition of the ethanolic fraction were analyzed to consider the possibility of its further valorization.

### 2.1. Modeling of the Acid Treatment Process

In order to optimize the acid treatment of seaweed biomass regarding the yield of isolated alginate, 17 experimental runs were planned according to the matrix of the Box–Behnken design (BBD); the conditions are presented in Table 1. Each experimental run represented a combination of different factor levels. The highest alginate yield related to the mass of dry biomass of 29.9% (experimental run 1) was achieved when the treatment time was 20 min, the liquid-to-solid ratio (the volume of hydrochloric acid per gram of used biomass after its depigmentation and defatting) was 10 mL/g, and the treatment temperature was 70 °C. The experimental run 15 had the lowest alginate yield of 9.4%, where the treatment time was 10 min, the liquid-to-solid ratio was 30 mL/g, and the treatment temperature was 55 °C.

The obtained data were fitted using a second-order polynomial model to achieve a better dependency between the analyzed factors and alginate yield. The polynomial equation that describes the extraction process in terms of coded values can be represented using Equation (1):

(1) $$Y=14.42+2.17A-2.71B-2.02C+6.43AB+AC-7.45BC+0.25A^{2}+6.67B^{2}+0.88C^{2}$$

where Y is the alginate yield and A, B, and C are the treatment time, liquid-to-solid ratio, and treatment temperature, respectively.

Regression coefficients are the first indicators of how and to what extent the terms in the polynomial equation impact the system response (alginate yield). The terms with positive regression coefficients lead to an increase in the alginate yield upon increasing their values and vice versa. The higher the values, the more they will affect the alginate yield. Based on the analysis of variance (ANOVA) presented in Table 2, the effect of terms on the alginate yield was estimated. The F-value can be used as a suitable parameter for that estimation [16]. The interaction between the liquid-to-solid ratio and treatment time had the highest F-value of 232.7, indicating that increasing this factor level will lead to a significant increase in the alginate yield. Among the linear terms, the highest negative impact had a liquid-to-solid ratio, while the low and almost identical impact was observed for the treatment time and temperature. 

Terms with *p*-values lower than 0.05 were considered statistically significant terms [16]. In this case, all terms were statistically significant except for the interaction AC and quadratic terms A^2^ and C^2^. The statistically non-significant terms are commonly excluded from a regression model to improve its prediction ability [17]. In addition to the lack-of-fit value, the coefficient of variation (CV) and adequate precision were used as measures for the estimation of model adequacy. The CV is also known as the relative standard deviation (RSD), of which the value is about 5.40% for the proposed model. The adequate precision suggests that the signal-to-noise ratio was higher than four, which represents a desirable value [18]. The adequate precision of 27.1 indicated that the obtained signal was adequate. The high correlation coefficient (R^2^ = 0.9908) implied a good agreement between experimental and predicted alginate yield values, i.e., the regression model could explain a 99.08% variation in the alginate yield. The difference between the coefficient of determination and the adjusted coefficient of determination was lower than 0.2, which is one more confirmation of the applied model’s adequacy [19].

**Figure 1:**
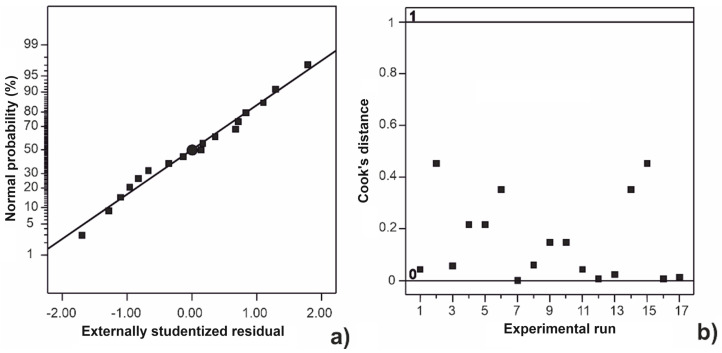
Normal distribution plot (**a**) and Cook’s distance (**b**) for the second-order polynomial model.

In Figure 1, the normal probability plot and Cook’s distance for the polynomial model are depicted. Since the points follow the straight line in the normal probability plot, it can be concluded that the data had a normal distribution of residuals. Cook’s distance is a useful tool to recognize the data that should be excluded from further analysis. The rule is to exclude the outliers, i.e., the data that overcome the limit value (1). In this study, the outliers were not observed, so there was no need to exclude the data on alginate yield during model generation and analysis. The further analysis was continued with a full quadratic model because the prediction ability was not improved by its reduction.

The BBD has the center point that represents the combination of mean levels of analyzed factors in the design space (factor level 0). This point (marked by the star in Table 2) is repeated a few times to obtain the statistical model parameters necessary for the estimation of its adequacy. Within the statistical analysis of the obtained data, the residual error was compared to pure error based on the lack-of-fit parameter. The F-value of lack-of-fit of 0.5 was not statistically significant compared to the pure error (4.7) because its value was lower than the critical F-value (F_crit_, 95% (3, 4) = 6.59).

**Figure 2:**
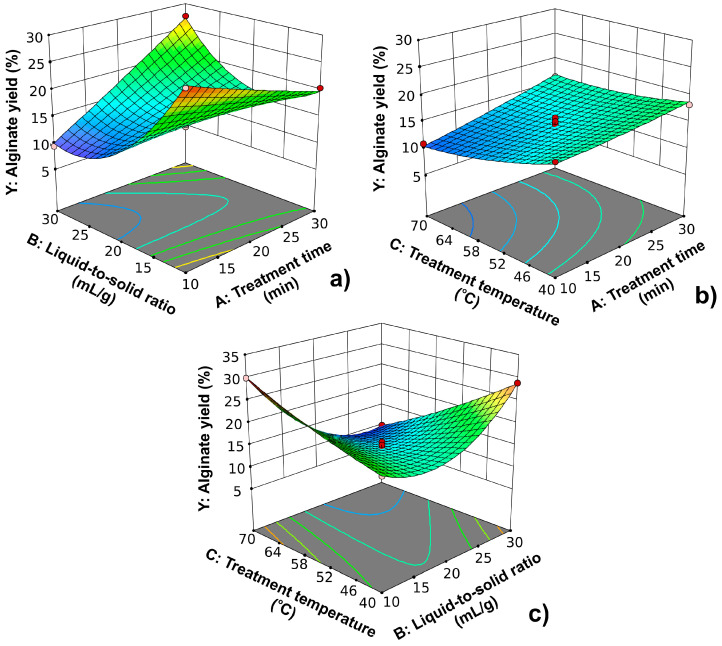
The effect of (**a**) treatment time and liquid-to-solid ratio at a treatment temperature of 55 °C; (**b**) the effect of treatment time and treatment temperature at a liquid-to-solid ratio of 20 mL/g; (**c**) the effect of liquid-to-solid ratio and treatment temperature for a treatment time of 20 min on alginate yield.

The three-dimensional plot representing the effect of treatment time and liquid-to-solid ratio on the alginate yield at a treatment temperature of 55 °C is shown in Figure 2a. The effect of the liquid-to-solid ratio was more pronounced at shorter treatment times. The alginate yield was decreased by increasing the liquid-to-solid ratio. With prolonging the treatment times, the alginate yields firstly decreased upon increasing the liquid-to-solid ratio until reaching its mean level and then again increased upon further increasing its factor level. By analyzing the obtained dependence, the decrease in alginate yield can be noticed due to the extension of the treatment time at lower liquid-to-solid ratios. At larger liquid-to-solid ratios, a sudden increase in the alginate yield occurred due to the extension of the treatment time. The strong interaction between these two observed parameters can be also noted based on the shape of the obtained plot, as shown in Figure 2a. The effect of treatment time and temperature on the alginate yield at a liquid-to-solid ratio of 20 mL/g is depicted in Figure 2b. In this case, a strong interaction between analyzed factors was not noticed. The interaction was a statistically non-significant term in the polynomial equation according to the results of the ANOVA test. The treatment time had a positive effect, while the temperature negatively affected the yield of alginate. In Figure 2c, the interaction between the liquid-to-solid ratio and treatment temperature at a treatment time of 20 min is presented. Also, a strong interaction between these factors was noticed. The treatment time had a positive effect on alginate yield at lower liquid-to-solid ratios, while it had a negative effect at higher levels. At shorter treatment times, there is an increase in the alginate yield due to the increase in the liquid-to-solid ratio. In the case of longer treatment times, the liquid-to-solid ratio had a negative effect on the alginate yield.

In general, the extension of treatment time leads to better contact between the solvent and plant material, that is, to a longer effect of ultrasounds. Due to the pressure difference, bubbles are created in places of lower pressure. Upon implosion, these bubbles generate microjets with significant energy, enabling the destruction of cell walls. The treatment temperature and treatment time are inter-related. The increase in temperature causes a decrease in the required time to achieve a successful extraction process. The volume of solvent must be sufficient to ensure the complete immersion of the plant material throughout the extraction process. There are various studies dealing with the evaluation of the effect of the liquid-to-solid ratio on the yield of the desired compounds. A high liquid-to-solid ratio is generally significant for conventional extraction techniques. The increase in the liquid-to-solid ratio significantly affects the increase in the solubility and diffusion coefficient of the compound [20]. A sufficiently large volume of solvent (hydrochloric acid) hydrolyzes and washes the desired component from the surfaces of damaged plant cells. In non-damaged plant cells, the solvent penetrates through the cell walls, dissolves (hydrolyzes) the desired compounds, and then diffuses back again. The concentration gradient is more significant at higher liquid-solid ratios, and in addition, there is better diffusion of the desired compounds [21].

### 2.2. Numerical Optimization of Acid Treatment of Brown Seaweed Biomass

After modeling and fitting the obtained data using the second-order polynomial model, the numerical optimization method of the response was carried out. As an optimization criterion, the maximum alginate yield was set. The following optimal conditions: a treatment time of approximately 30 min, a liquid-to-solid ratio of approximately 30 mL/g, and a treatment temperature of approximately 47 °C were obtained for acid treatment under the effect of ultrasound. At the suggested optimal extraction conditions, the proposed regression model predicted an alginate yield of 31.3%. Under the same extraction conditions, the acid treatment of brown seaweed was carried out to estimate the prediction ability of the model. The experimentally obtained alginate yield was found to be 30.9%, which was very close to the predicted value. Bearing in mind that there is a minimal difference between the experimental and predicted alginate yields, the regression model can be suitable for the prediction of alginate yield. Also, it is important to point out that the acid treatment time in this study was many times shorter than the times necessary for the conventional procedure (maceration), probably due to the effect of ultrasounds. Trica et al. [10] isolated alginate from brown seaweed *Cystoseira barbata* (the Romanian coast on the Black Sea) with a yield of 19% (*w*/*w*) under the following acid treatment conditions: 0.1 mol/L HCl at 60 °C for 2 h. By comparing this result with those obtained in this study, it can be concluded that the acid treatment of biomass supported by ultrasound enabled a more efficient process in terms of the alginate yield and a reduction in necessary treatment time.

### 2.3. Fourier-Transform Infrared Analysis

**Figure 3:**
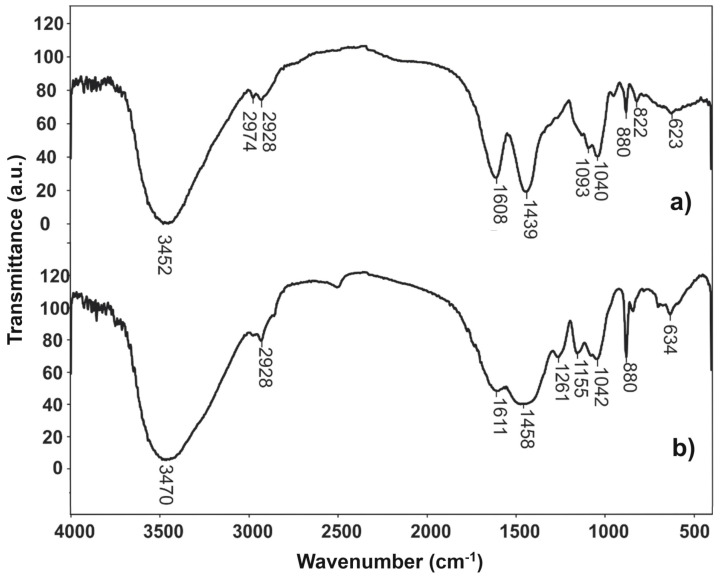
FTIR spectra of commercial alginate (**a**) and isolated alginate (**b**) from brown seaweed *L. digitata*.

The alginate surface chemistry and identification of functional groups were achieved by comparison of the Fourier-transform infrared (FTIR) spectra of alginate isolated from brown seaweed under the optimal conditions of UAE with commercial alginate. In the spectrum of commercial alginate (Figure 3a), the characteristic bands were identified at 3452 cm^−1^ (valence vibration of the O–H group), 2974 and 2928 cm^−1^ (valence vibrations of the sp^3^ C–H bond), 1608 cm^−1^ and 1439 cm^−1^ (asymmetric and symmetric valence vibration of the COO^−^ group, respectively), 1093 cm^−1^ (OH bending, C–C stretching of pyranose belonging to guluronic acid), 1040 cm^−1^ (C–O–C valence vibration of mannuronic acid), and 880 cm^−1^ (the C_1_–H deformation of mannuronic acid). In the FTIR spectra of isolated alginate (Figure 3b), the same bands were noticed at 3470, 2928, 1611, 1458, 1079, 1042, and 880 cm^−1^ but with slight shifts in the wavenumbers. Beratto et al. [22] also confirmed the presence of these characteristic bands in alginate molecules so that the proposed procedure led to the efficient isolation of alginate from brown seaweed. The ratio of *β*-d-mannuronic (M) and *α*-l-guluronic (G) acids in the sample was estimated based on the ratio of absorbance at around 1040 and 1090 cm^−1^. In this study, the M/G ratio for isolated alginate was 1.08, indicating the higher presence of M-blocks compared to G-blocks. Alginates with such structures can form higher viscosity solutions, as well as soft and elastic gels, than those with higher contents of G-blocks. This ratio can vary depending on the extraction procedure, the kind of seaweed, and its growth location. In the literature [22], these values ranged between 1.056 and 1.164 for two Chilean brown seaweeds, *Lessonia spicata* and *Macrocystis pyrifera*. Fertah et al. [23] obtained the M/G ratio of 1.12 for alginate isolated from Moroccan *L. digitata* via the conventional extraction technique (acid treatment of biomass with 0.2 mol/L during 24 h). These results indicate that the obtained M/G ratio agrees with the data available in the literature, even though the seaweed species and extraction procedure are different from each other. 

### 2.4. Chemical Characterization of the Ethanolic Fraction of Brown Seaweed Biomass

#### 2.4.1. Total Antioxidant Content and Total Flavonoid Content

Antioxidants in brown seaweed are present as a chemical defense mechanism of the plant itself; otherwise, they are a large group of compounds of different compositions and structures. In the residual ethanolic fraction of brown seaweed examined in the present study, the total antioxidant content (TAC) was 293 mg GAE/g dry weight (milligrams of gallic acid equivalents per gram of dry weight). The GAE was used to express the TAC for easier comparison of the obtained experimental data in this study with those obtained in the other studies. In the literature, the TAC was determined in the extracts of the *L. digitata* seaweed from different territories obtained under different UAE conditions. For instance, Wekre et al. [24] obtained the TAC of 6.860 mg GAE/g d.w. for a 50% (*v*/*v*) ethanolic extract of seaweed (Melbourne, Victoria); D’Este et al. [25] determined the TAC of 47.400 mg GAE/g d.w. for a 60% (*v*/*v*) ethanolic extract of seaweed (Denmark), while Heffernan et al. [26] and Ummat et al. [27] determined the TACs of 0.002 mg GAE/g d.w. and 72.600 mg GAE/g d.w. for the 80% (*v*/*v*) and 50% (*v*/*v*) ethanolic extracts of seaweed (Ireland), respectively. After analysis, it was concluded that the TAC of the residual ethanolic fraction of brown seaweed is significantly higher than those available in the literature. The difference in TAC can be attributed to the geographical location of the seaweed, harvesting season, drying method, solvent concentration used, extraction technique, and conditions. Even though the UAE is an efficient technique, the maceration at room temperature is presented here as the method that gives the ethanolic fraction with an extremely high TAC.

The obtained total flavonoid content (TFC) of 14.9 mg RE/g d.w. (rutin equivalents per gram of dry weight) was difficult to compare with other available results because the content was mainly expressed in terms of quercetin equivalents (QE). Otherwise, Ummat et al. [27] reported a TFC of 15.2 mg QE/g in a 50% (*v*/*v*) ethanolic extract of *L. digitata* obtained via UAE.

#### 2.4.2. Identification of Extracts’ Chemical Constituents via the Chromatographic Method

**Figure 4:**
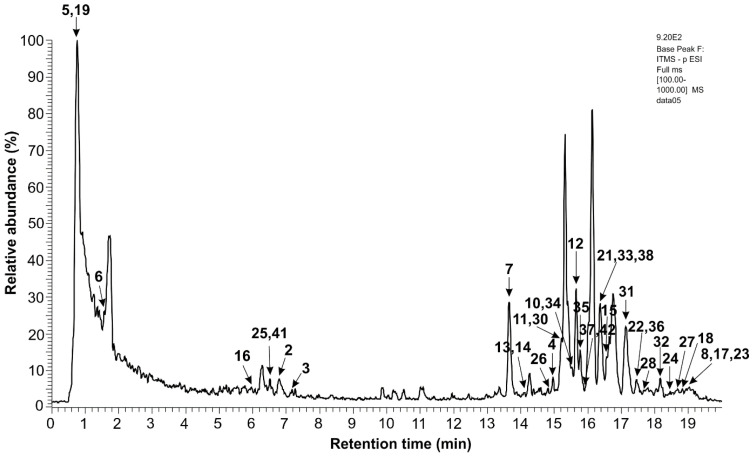
The base peak chromatogram of the ethanolic fraction of brown seaweed (*m*/*z* 100–1000). The numbers in the chromatogram refer to the identified compounds whose nomenclatures are presented tabularly.

The identification of the extracts’ chemical constituents of brown seaweed was carried out using ultra high-performance liquid chromatography–electrospray ionization–tandem mass spectrometry (UHPLC-ESI-MS/MS) in negative mode. The chromatogram obtained according to the base peak in the mass range of *m*/*z* 100–1000 is presented in Figure 4. In Table 3, there are 48 detected compounds, of which seven compounds were not identified. The analysis confirmed that the extract contains sugar alcohol, phenolic compounds, and lipids.

Sugar alcohol. Mannitol was identified at t_R_ = 0.86 min with [M − H]^−^ ion at *m*/*z* 180.90 (**1**). This compound belongs to the group of sugar alcohols, the members of which are most widely used as sweeteners for diabetic food products and medication (osmotic diuretic) [42]. Premarathna et al. [43] reported that the aqueous extract of brown seaweed *Sargassum ilicifolium*, which also contains D-mannitol, has the potential property of healing skin wounds.

Phenolic compounds. Polyphenols represent one of the most abundant and widespread plant secondary metabolites, with over 8000 polyphenolic structures. They are strong natural antioxidants, which protect and strengthen plants against the harmful effects of UV radiation, persistent insects, and extreme weather conditions [44]. In humans, phenolic compounds play an important role in strengthening immunity, metabolism, recovery from inflammation, improving mood, and preventing and treating chronic diseases, such as cardiovascular disease, diabetes, neurodegenerative disorders, and cancer [45]. Considering these positive effects on people’s health, it is essential to use them daily. In the analyzed extract, phenolics including phenolic compounds, phenolic acids, stilbenes, flavonoids, and tannins were the most abundant among the identified compounds.

Phenolic acids and their derivatives. In the extract, the different phenolic acids in the pure and conjugated forms were identified. The glucoside form of monohydroxybenzoic acid (**3**) had a peak at t_R_ = 7.20 min with an [M − H]^−^ ion at *m*/*z* 260.95. The peak with an [M − H]^−^ ion at *m*/*z* 304.87 and fragment ions at *m*/*z* 175 (100%) and 131 originated from 2-isopropylmalic acid (**8**). The compound with an [M − H]^−^ ion at *m*/*z* 311.22 (t_R_ = 15.48 min) and characteristic fragment ions at *m*/*z* 149 (100%) was identified as caftaric acid (10), while the [M − H]^−^ ion at *m*/*z* 378.69 (t_R_ = 18.89 min) had its derivative (18). The peak with an [M − H]^−^ ion at *m*/*z* 317.03 (t_R_ = 15.11 min) indicated a *p*-coumaric acid derivative (**11**). 5-*O*-*p*-Coumaroyl shikimic acid (**12**) gave an [M − H]^−^ ion at *m*/*z* 319.18, which further successively produced ions at *m*/*z* 301 (100%), 275, 257, 203, 179, 167, and 115. Coumaroylglucaric acid isomer (**15**) had an [M − H]^−^ ion at *m*/*z* 355.30 (t_R_ = 16.54 min). 1-Caffeoylquinic acid (**14**), which had an [M − H]^−^ ion at m/z 353.21, occurred at t_R_ = 14.20 min. It produced characteristic fragment ions at *m*/*z* 335, 317, 310, 256, 191 (100%), 173, and 123. Three other phenolic acid derivatives were also detected, including three caffeic acid derivatives (**34**, **35**, and **36**). Zhong et al. [46] also confirmed the presence of these phenolic acids and their derivatives in different seaweeds. Otherwise, this group of phenolic compounds can be useful for improving human health due to its members’ expressed antioxidant properties [47].

Stilbene. Resveratrol (**5**), as a representative of stilbene, formed a formate adduct [M − H + HCOOH]^−^ ion at *m*/*z* 272.92 (t_R_ = 0.65 min) and characteristic fragment ions at *m*/*z* 227, 217, 159 (100%), and 115. Wu et al. [48] confirmed the presence of this compound in the seaweed extracts, including the alcoholic extract of *Sargassum polycystum* from the South China Sea.

Flavonoids and their derivatives. The following flavonoid classes were detected in the analyzed extract: flavanols, flavones, flavonols, flavanonols, and flavanone. From the subclasses of flavonoids, two different flavanol derivatives (**23** and **27**) were identified. The peaks with [M − H]^−^ ions at *m*/*z* 440.87 (**23**) and 508.70 (**27**) were identified as epigallocatechin derivatives. The presence of various flavonols was confirmed in the extract, i.e., isorhamnetin derivative (**13**, [M − H]^−^ ion at *m*/*z* 351.03), quercetin derivatives (**19** and **25**, [M − H]^−^ ions at *m*/*z* 379.12 and 484.95, respectively), kaempferol derivative (**26**, [M − H]^−^ ion at 501.12), kaempferol-3-*O*-glucoside (**28**, [M − H]^−^ ion at 520.65), and rutin (**41**, [M − H]^−^ ion at *m*/*z* 609.02). Compounds **2**, **30**, **31,** and **38** were identified as flavones. The peak with an [M − H]^−^ ion at *m*/*z* 236.93 originated from 7-hydroxyflavone (**2**), while the peak with an [M − H]^−^ ion at *m*/*z* 527.30 was due to the presence of the sulfate of orientin (**30**). Luteolin derivative (**31**) had a peak with an [M − H]^−^ ion at *m*/*z* 547.40. Hydroxyisoflavone 3-*O*-methylorobol derivative (**38,** [M − H]^−^ ion at *m*/*z* 581.30) was also identified in the extract, while the [M − H]^−^ ion at *m*/*z* 549.45 (t_R_ = 18.16 min) originated from flavanone liquiritigenin-hexose-xyl/ara (**32**).

Flavonoids and their derivatives are considered among the most abundant phenolic compounds in different types of seaweed [48,49]. These compounds exhibit various biological activities and can be used in the prevention of many human diseases [50].

Tannins and their derivatives. A special type of tannins, which are exclusively synthesized by brown seaweed to protect them from stress conditions, such as UV radiation and herbivorous species, are phlorotannins [41]. Gheda et al. [51] confirmed that phlorotannins have antioxidant, anti-inflammatory, antidiabetic, and other biological activities. It is worth noting that there are many different types of phlorotannins, with different molecular weights and structures, so it is not easy to identify a specific compound based on the mass spectrum alone. Fucophlorethol (**17**) was confirmed based on an [M − H]^−^ ion at *m*/*z* 372.83 (t_R_ = 18.91 min) and fragment ion at *m*/*z* 305. Compounds **16**, **20**, **21**, **24**, and **45** with [M − H]^−^ ions at *m*/*z* 360.93, 384.77, 385.20, 452.73, and 723.10 were identified as phlorotannin derivatives, among which compound **45** was a sulfate derivative.

Lipids. Certain lipid classes have been identified in the extract, including sterol lipids, polar lipids, and fatty acids.

Sterol lipid. Similar to the previous study [30], sterol lipid (**22**), which had an [M − H]^−^ ion at *m*/*z* 439.29, was also detected in the analyzed extract.

Polar lipids. Among the polar lipids, glycolipids (**33**, **37**, and **44**), phospholipid (**42**), and phosphatidylglycerol (**43**) were identified in the extract. Two different monoacylglycerols in conjugation with a sulfoquinovosyl part (**33** and **37**), which differ from each other in terms of the number of carbon atoms and double bonds in the fatty acid side chains, were detected at t_R_ = 16.27 min and 15.90 min with [M − H]^−^ ions at *m*/*z* 555.32 and 579.25, respectively. Another glycolipid (**46**) was detected at t_R_ = 15.50 min with an adduct [M + HCOO]^−^ ion at *m*/*z* 721.15. Phosphatidic acid (C:N 34:4), as an anionic phospholipid (**42**), was also identified. Phosphatidylglycerol (C:N 32:1) (**43**) was identified based on an [M − H]^−^ ion at *m*/*z* 719.9. de Costa et al. [52], for these types of polar lipids, identified in a red seaweed *Grateloupia turuturu*, showed that health benefits are due to the antioxidant and anti-inflammatory properties. In a review study, Arif et al. [53] stated that triacylglyceride is convertible to biodiesel. In contrast, glycolipids and phospholipids cannot be converted to biodiesel due to their high contents of polyunsaturated acids, making the production process insufficient and expensive.

Fatty acids. Octadecatrienoic acid with an [M − H]^−^ ion at *m*/*z* 277.01 (**6**) was identified at t_R_ = 1.56 min, while its hydroxide with an [M − H]^−^ ion at *m*/*z* 293.04 was detected at t_R_ = 13.59 min (**7**). The presence of the same fatty acids was also confirmed in the methanolic extracts of the red seaweed species *Turbinaria triquetra* and *Polycladia myrica* [30]. Oxidized fatty acid (**4**) (t_R_ = 14.94 min) with an [M − H]^−^ ion at *m*/*z* 265.12 was also present in the analyzed extract.

#### 2.4.3. Determination of the Concentrations of Macroelements and Microelements within the Studied Fraction

The contents of macroelements and microelements present in the ethanolic fraction of brown seaweed were determined using ICP-OES. The concentrations of various essential macroelements (Ca, K, Mg, Na, P, S, and Si) and microelements, including heavy metals (Al, B, Ba, Cd, Co, Cr, Cu, Fe, Li, Mn, Ni, Pb, Sr, Zn), are presented in Table 4. The most abundant macroelement was Na, with a content of 106.59 ± 1.23 mg/g d.w. Moreover, the extract exhibited high contents of K, S, and Mg, while the contents of other detected macroelements were significantly lower, ranging between 0.03 mg/g d.w. and 4.57 mg/g d.w. Also, it can be noticed that boron (B), iron (Fe), manganese (Mn), and zinc (Zn) emerged as the most prevalent microelements in the extract. Other detected microelements were present at contents lower than 1.93 μg/g d.w. Notably, the total content of microelements in the ethanolic fraction of brown seaweed exceeds that of macroelements by approximately 34%. In the extract, the sodium-to-potassium (Na-to-K) molar ratio is very important because it significantly impacts the cardiovascular system. An intake of sodium of less than 2000 mg/day and potassium of more than 3510 mg/day is recommended according to the World Health Organization [54]. In this extract, the determined Na-to-K molar ratio of 4.92 was too high, so people should be careful during its intake. Singh et al. [55] obtained a similar Na-to-K ratio of 4.85 for the seaweed species *Gracilaria edulis*, while the seaweed species *Kappaphycus alvarezii* had a different Na-to-K ratio of 0.01. They also confirmed the presence of iron (12.67 × 10^3^–86.1 × 10^3^ μg/L), manganese (2.1 × 10^3^–125.8 × 10^3^ μg/L), nickel (0.212 × 10^3^–3.45 × 10^3^ μg/L), copper (0.044–0.65 × 10^3^ μg/L), zinc (0.628–4.7 × 10^3^ μg/L), lead (17.4 × 10^3^ μg/L), and chromium (0.004–32.0 × 10^3^ μg/L) among the identified microelements. That study concludes that adding 2.5% each of *Kappaphycus alvarezii* or *Gracilaria edulis* sap can reduce 50% of the fertilizer needs of the crop during foliar use. 

### 2.5. Biological Activities of the Ethanolic Fraction of Brown Seaweed Biomass

#### 2.5.1. Radical Scavenging Activity

**Figure 5:**
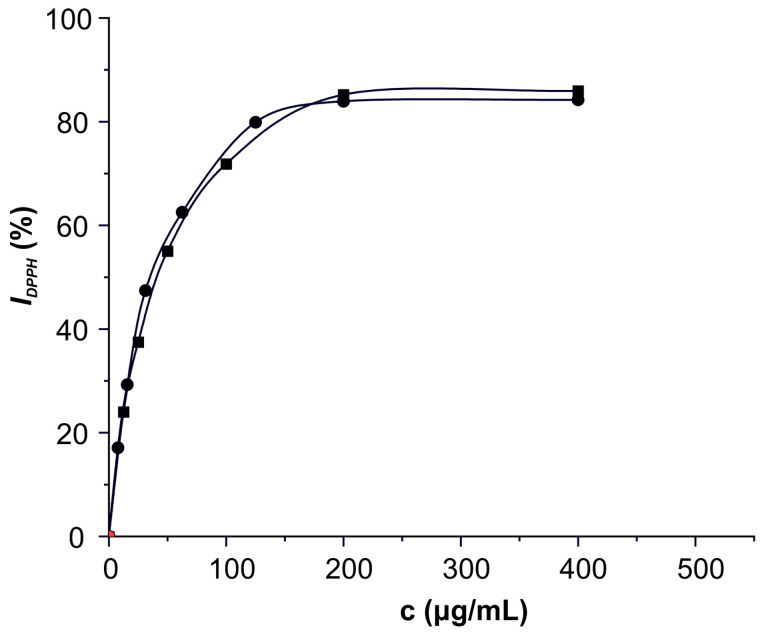
The dependencies of DPPH radicals’ inhibition (*I_DPPH_*) on the concentrations (c) of seaweed extract (■) and the synthetic antioxidant butylhydroxytoluene (●) after incubation for 30 min.

The 2,2-diphenyl-1-picrylhydrazyl (DPPH) assay was used to determine the radical scavenging activity of the ethanolic fraction of brown seaweed biomass. The inhibitory effect of DPPH oxidation as a function of increasing the sample concentration is presented in Figure 5. The obtained profiles of the analyzed extract and the synthetic antioxidant butylhydroxytoluene (BHT) are almost identical, which implies that they have a similar ability to inhibit DPPH free radicals even up to 85%. Based on the half-maximal inhibition concentration (IC_50_ value) of 42.84 ± 0.81 μg/mL, the extract in our study expressed pronounced radical scavenging activity, being about 18% lower than the activity of the synthetic antioxidant, typically applied in the food industry (IC_50_ = 36.61 ± 0.79 μg/mL). Ummat et al. [27] concluded that phenolic compounds significantly contribute to the radical scavenging activity of the seaweed extract. With such pronounced radical scavenging activity, the extract of brown seaweed biomass could be used as a source of natural antioxidants.

The *L. digitata* extract has a high radical scavenging activity, which is significantly influenced by the technique for the preparation of the extract. In the study of Ummat et al. [27], the 50% (*v*/*v*) ethanolic extracts of different types of seaweed obtained via UAE had higher radical scavenging activities compared to the extracts obtained via a conventional extraction procedure (at 20 °C for 4 h). Furthermore, the authors concluded that among all 11 analyzed seaweed species, *L. digitata* showed the lowest radical scavenging activity. Intercomparison of the IC_50_ value of the extract prepared in this study with the available literature data was difficult due to differently expressed units and various extraction conditions.

#### 2.5.2. Antimicrobial Activity of Ethanolic Fraction of Brown Seaweed Biomass

The antimicrobial activity of the alginate extraction byproduct (ethanolic fraction) was tested against several foodborne pathogenic bacteria and one yeast strain. As presented in Table 5, the growth of all tested bacterial strains was inhibited by the presence of the ethanolic fraction of brown seaweed biomass. In most of the cases, the concentration required for bacterial growth inhibition was 10 mg/mL, except for *S. aureus* and *B. cereus*, in which cases 5 mg/mL was sufficient to inhibit microbial growth. Similarly, Jassbi et al. [56] tested several extracts derived from red and brown algae and found that they expressed the highest antimicrobial activity toward *S. aureus* and *B. subtilis*. The authors identified free fatty acids as responsible for the antimicrobial activity of their extracts. Horincar et al. [57] reported the same group of compounds as responsible for the antimicrobial activity of extracts of macroalgae from the Romanian Black Sea coast against the same bacteria. In our study, free fatty acids were also identified as constituents of ethanolic byproducts. *S. aureus* was the most susceptible to ethanolic seaweed extracts (74% of all tested samples) in the study reported by Padmakumar and Ayyakkannu [58]. In the same study, *P. aeruginosa* was the most resilient, and only a few extracts suppressed its growth. The ethanolic fraction from our study not only inhibited this bacterium but was also able to kill it. 

Gram-positive bacteria were more susceptible to the seaweed biomass byproduct, which is in line with the findings of Salem et al. [59]. Interestingly, the ethanolic fraction exhibited a microbicidal effect toward all bacterial strains tested in our study in a concentration range of 10–20 mg/mL. Gentamicin, as a positive control, was effective in significantly lower concentrations, except in the case of *S. aureus*, where no minimum bactericidal concentration (MBC) was recorded. This is an important observation since the ethanolic fraction is a naturally derived product, as well as a byproduct with no commercial value at the moment. Hellio et al. [60] tested the antimicrobial activity of aqueous, ethanolic, and dichloromethane extracts from 30 marine algae species (including *L. digitata*) against more than 30 marine bacteria and reported that about 20% of them were active. In this study, the minimum inhibitory concentration (MIC) ranged from 24 to 96 μg/mL which is around 50–200 times lower MIC than in our study. However, the species tested were different, as were the types of extracts, and in our case, a byproduct instead of crude or purified extract was evaluated. On the other hand, Boisvert et al. [61] tested the antimicrobial activities of several edible seaweed species’ ethanol extracts against food spoilage bacteria (including *E. coli*). The authors applied microdilution assay and reported dose-dependent antimicrobial activity, which was the highest against *E. coli*. However, some of the samples tested in their report were more effective against Gram-positive bacteria. Overall, it is impossible to efficiently compare the existing literature data due to differences in antimicrobial assays’ methodologies, algal species, techniques of extract preparation, and the way the results were expressed.

The ethanolic fraction in our study suppressed the growth of pathogenic yeast at the highest tested concentration and did not show fungicidal activity. Several other authors reported antifungal activity toward *C. albicans* when different types of extracts from several macroalgal species were tested [62,63].

A relatively high antimicrobial activity of the ethanolic fraction obtained as a byproduct of alginate extraction was reported, especially its microbicidal effect against several foodborne pathogens, making it a potentially highly valuable antimicrobial compound. Still, more research is needed to evaluate this byproduct to classify it as *generally recognized as safe* (GRAS), thus being of importance for the food and feed industry. Moreover, antimicrobial activity was more expressed against Gram-positive species, a prerequisite for products intended as antifouling agents, applicable in aquaculture [64].

## 3. Materials and Methods

### 3.1. Chemicals and Plant Material

In this work, 96% (*v*/*v*) ethanol, sodium carbonate, methanol, formic acid (Zorka Pharma, Šabac, Serbia), the Folin–Ciocalteu reagent, gallic acid (97%), 2,2-diphenyl-1-picrylhydrazyl (DPPH), HPLC-MS-grade water and acetonitrile (Sigma Chemical, St. Louis, MO, USA), and alginic acid sodium salt with very low viscosity (pH 5.68 and dynamic viscosity of 37.8 mPa·s) (Thermo Scientific^TM^, Waltham, MA, USA) were used. All other chemicals used were of analytical grade, purchased from Sigma-Aldrich (Saint-Louis, MO, USA). Tryptic soy agar, tryptic soy broth (TSB), malt broth, malt agar, 2,3,5-triphenyl tetrazolium chloride, nystatin, and Gentamicin were procured from Sigma Chemical Co., (St. Louis, MO, USA).

The brown seaweed (*Laminaria digitata*, Laminariaceae) biomass was purchased from Ozon Way (Belgrade, Serbia). The material was dried at room temperature for a week in the dark to prevent possible degradation of bioactive compounds. After that, the dry biomass was ground in an electric mill (Braun Aromatic KSM2, Kronberg im Taunus, Germany) and then sieved. A fraction of 0.5 mm was used for the extraction of alginate from biomass. The biomass moisture content of 10.6% (*m*/*m*) was determined gravimetrically by drying the sample at 105 °C in a laboratory oven.

### 3.2. Alginate Extraction from Brown Seaweed

Alginate extraction was carried out according to the procedure described in the literature [10], with minor modifications, involving the following 5 steps: the depigmentation and defatting of biomass with ethanol, acid treatment with hydrochloric acid, alkaline treatment with sodium carbonate, precipitation with ethanol, and the drying of alginate. Precisely, the dried brown seaweed biomass (500 g) was first subjected to depigmentation and defatting with 70% (*v*/*v*) ethanol (5 L). After 24 h, the biomass was separated from the solvent via vacuum filtration on a Buchner funnel. The seaweed biomass was further ultrasonically treated using an ultrasonic bath (operating frequency of 40 kHz and power of 150 W) (Sonic, Niš, Serbia) in the presence of 0.1 mol/L hydrochloric acid. The obtained sample was subjected to vacuum filtration, during which the biomass was washed with distilled water to remove excess acid. In the next step, the biomass was extracted with a 3% (*m*/*v*) Na_2_CO_3_ at a liquid-to-solid ratio of 20 mL/g and a temperature of 60 °C for 2 h. Before centrifugation (3000 rpm, 15 min), the extracts were cooled to room temperature. The alginate precipitation from the resulting solution was performed by adding 96% (*v*/*v*) ethanol. After the determination of the optimal conditions for the acid treatment of biomass with hydrochloric acid supported by ultrasounds, bleaching of sodium alginate was performed twice with 20 mL of acetone at 18 °C. The obtained sodium alginate was dried at 50 °C, ground in a laboratory mill, and sieved through a 0.2 mm sieve.

### 3.3. Optimization of Acid Treatment of Biomass 

The acid treatment of biomass supported by ultrasound was modeled and optimized using the BBD. In the first part of this research, the effects of treatment time, liquid-to-solid ratio, and treatment temperature on the alginate yield were analyzed during the optimization study. The response surface methodology was applied to obtain the optimal levels of the analyzed factors, based on a smaller number of performed experiments. The BBD was used to plan the extraction conditions. It represents a combination of fractional factorial and incomplete block design, so the analysis was carried out by avoiding the surfaces in extreme values as in the central composite design [65]. Also, one of the essential characteristics of BBD is its sphericity. In other words, this model does not contain combinations of parameters in which they are all at their lowest or highest levels. Experimentally obtained data at 3-level BBD are used to obtain a quadratic relationship between input factors and response using non-linear regression analysis. Based on this approach, a mathematical model was obtained that could predict the behavior of the analyzed response in the design space.

The treatment time (10–30 min), liquid-to-solid ratio (10–30 mL/g), and treatment temperature (40–70 °C) were analyzed as factors (independent variables), while the alginate yield was observed as the system response (dependent variable). The analyzed factor levels (Table 6) were analyzed according to our previous knowledge about the ultrasound-assisted extraction process and the already-described procedure of the acid treatment of biomass [1].

For better uniformity, factor-level values were transformed into coded values according to Equation (2):

(2) $$x_{i}=\frac{X_{i}-X_{o}}{∆X_{i}}$$

where *x_i_* is the coded value, *X_i_* is the actual value of the factor, *X_o_* is the actual value at the center point, and Δ*X_i_* is the step-change value.

The obtained data for the yield of sodium alginate were fitted using a second-order polynomial equation, which, in its general form, can be presented as follows (Equation (3)):

(3) $$Y=a_{0}+a_{1}x_{1}+a_{2}x_{2}+a_{3}x_{3}+a_{11}x_{1}^{2}+a_{22}x_{2}^{2}+a_{33}x_{3}^{2}+a_{12}x_{1}x_{2}+a_{13}x_{1}x_{3}+a_{23}x_{2}x_{3}+\epsilon$$

where Y is the system response; *a*_0_ is the intercept; *a*_1_, *a*_2_, *a*_3_, *a*_11_, *a*_22_, *a*_33_, *a*_12_, *a*_13_, and *a*_23_ are the regression coefficients; *x*_1_, *x*_2_, and *x*_3_ are the factors; and *ε* is the residual.

### 3.4. Determination of Isolated Alginate Surface Chemistry

The FTIR was used to record the spectra of standards and isolated alginate after the preparation of KBr pellets. Recording of the samples was carried out in the wavenumber range of 4000–400 cm^−1^ with a resolution of 2 cm^−1^ on a Bomem Hartmann and Braun MB-series spectrophotometer (Quebec, QC, Canada). The spectra were processed using Win-Bomem Easy software (Bomem GRAMS/32, Galactic Industries, Salem, NH, USA). The absorbances at around 1040 cm^−1^ and 1090 cm^−1^ were measured to calculate the M/G ratio of isolated alginate and estimate its quality.

### 3.5. Chemical Characterization of the Ethanolic Fraction of Brown Seaweed Biomass

#### 3.5.1. Determination of the Total Antioxidant Content

The ethanolic fraction of brown seaweed biomass that represents waste after alginate isolation was subjected to chemical analysis to assess the possibility of its further use. The TAC of the ethanolic fraction was determined spectrophotometrically using the Folin–Ciocalteu reagent [66]. Otherwise, the TAC is a more desirable term than total phenolic content because phenolic compounds are not the only compounds that can reduce the Folin–Ciocalteu reagent. Considering this fact, a wrong conclusion can be derived that more phenolic compounds are present in the extract than truly exist. The sample was prepared by adding 1 mL of a ten-fold diluted aqueous solution of the Folin–Ciocalteu reagent and 1 mL of 7% (*m*/*v*) Na_2_CO_3_ into 0.1 mL of the extract. The sample was incubated for 90 min. The absorbance at 765 nm was measured on a Varian Cari-100 spectrophotometer (Mulgrave, Victoria, Australia). The TPC is expressed as mg GAE/g d.w.

#### 3.5.2. Determination of the Total Flavonoid Content

The TFC of the ethanolic fraction was determined via an indirect spectrophotometric method with aluminum(III)-chloride [67]. The sample for analysis was prepared by mixing 1.5 mL of 96% (*v*/*v*) ethanol, 0.1 mL of 10% (*v*/*v*) aluminum(III) chloride, 0.1 mL of 1 mol/L potassium acetate, and 2.8 mL of distilled water. Instead of aluminum (III) chloride, a blank sample contained an equivalent amount of distilled water. The absorbance of the samples was measured at 415 nm after 30 min of incubation at room temperature. The TFC is expressed as mg RE/g d.w.

#### 3.5.3. UHPLC-ESI-MS/MS Analysis

An UHPLC-ESI-MS/MS spectrometer (Dionex Ultimate 3000 UHPLC+) equipped with a quaternary pump, degasser, diode array detector (DAD), and mass detector (LCQ Fleet Ion Trap, Thermo Fisher Scientific, San Jose, CA, USA) was used for the qualitative analysis of bioactive compounds in the ethanolic fraction that remained after alginate isolation. The instrument control, data acquisition, and processing were carried out using Xcalibur (version 2.2 SP1.48) and LCQ Fleet (version 2.1, Thermo Fisher Scientific, San Jose, CA, USA) software. A Hypersil gold C18 column (50 mm × 2.1 mm, 1.9 μm) was used to separate the present compounds. The system was thermostated at 25 °C, while the flow rate of the mobile phase was 0.25 mL/min. The mobile phase consisted of phase A (0.1% formic acid in water) and phase B (0.1% formic acid in acetonitrile). The following linear gradient was applied for elution: 0–5 min (5–95% B), 5–6 min (95–5% B), and 5% B during the next 3 min. The DAD detector was adjusted to wavelengths of 210, 280, 310, and 560 nm. The mass spectra were recorded in negative mode, and the mass ranged from *m*/*z* 100 to 1000. The following ion source conditions were used for the analysis: a source voltage of 5 kV, a capillary voltage of 49 V, a tube lens voltage of 115 V, a capillary temperature of 275 °C, and a gas flow of the sheath and an auxiliary gas (N_2_) of 42 and 11 (arbitrary units). To study the fragmentation of the obtained molecular ions in the ion source, data-dependent scanning was carried out at a collision energy (“Collision Induced Dissociation”—CID) of 35 eV in a helium stream. The identification of the compounds was carried out based on a comparison of the mass spectra and retention times obtained in this study for various compounds with those available in the literature.

#### 3.5.4. ICP-OES Analysis of Ethanolic Fraction

The concentrations of elements in the tested ethanolic fraction were quantified using inductively coupled plasma optical emission spectrometry, ICP-OES (Thermo Scientific iCAP 6500 Duo ICP, Thermo Fisher Scientific, Cambridge, UK). Calibration solutions for ICP-OES measurements were prepared using three multi-elemental plasma standard solutions: Multi-Element Plasma Standard Solution 4, Specpure^®^, 1000 μg/mL (Alfa Aesar GmbH and Co. KG, Karlsruhe, Germany), ILM 05.2 ICS Stock 1, and SS-Low Level Elements ICV Stock (VHG Labs, Inc- Part of LGC Standards, Manchester, NH 03,103 USA). For each digested formulation, the ICP-OES measurements were conducted in triplicate. The reliability of measurements was validated via a relative standard deviation lower than 0.5%. The reported results are expressed in mg/L for macroelements and μg/L for microelements.

### 3.6. Biological Activities of the Ethanolic Fraction of Brown Seaweed Biomass

#### 3.6.1. Radical Scavenging Activity

The radical scavenging activity of the ethanolic fraction of brown seaweed was determined and compared with BHT using the DPPH assay [68]. Firstly, the dry weight content of the analyzed extract was calculated based on drying 3 mL of the sample to a constant mass at 105 °C in the laboratory oven. Exactly 2.5 mL of the extract was treated with 1 mL of a methanolic solution of DPPH radicals (0.3 mmol/L). The absorbance of the sample was measured at 517 nm after 30 min of incubation in the dark at room temperature. Instead of the ethanolic fraction, the positive and negative blank solutions contained the equivalent volume of BHT solution and pure methanol. The DPPH radical’s inhibition (*I_DPPH_*) expressed as a percentage was calculated according to Equation (4):

(4) $$I_{DPPH}\left(\%\right)=\frac{A_{C}-A_{S}}{A_{C}}\times 100$$

where *A_S_* is the absorbance of the sample treated with DPPH solution and *A_C_* is the absorbance of the negative control solution.

The radical scavenging activities of the samples were evaluated based on the IC_50_ value obtained via interpolation from the dependence between the DPPH inhibition and sample concentration.

#### 3.6.2. Antimicrobial Activity of the Ethanolic Fraction of Brown Seaweed Biomass

The antibacterial activity of the byproduct of alginate extraction was tested in triplicate on *Staphylococcus aureus* ATCC 6538, *Enterococcus faecalis* ATCC 29219, *Bacillus cereus* ATCC 10876, *E. coli* ATCC 25922, *Pseudomonas aeruginosa* ATCC 27853, and *Shigella sonei* ATCC 29930. The stock of bacterial strains was recovered via cultivation in TSB, and the starting concentration was set up based on the optical density corresponding to 10^6^ CFU/mL. The MIC and MBC concentrations were determined using the microdilution method, as descried by Vasiljevic et al. [69]. A 50 μL aliquot of the ethanolic fraction of brown seaweed biomass prepared in TSB in the range of concentrations, 0.156–20 mg/mL, was added to sterile flat-bottom 96-well microtiter plates (Sarstedt, Germany), inoculated with bacterial strains cultured in TSB, and incubated for 24 h. The MIC and MBC were determined with Gentamicin as a positive control and bacterial dilutions in TSB as a negative control, while TSB alone served as a sterility control.

### 3.7. Statistical Analysis

All measurements were carried out three times. The generation of regression models, ANOVA tests, three-dimensional plot presentations of dependency between alginate yield and factors, and other statistical analyses was performed using Design Expert 13 software (Stat-Ease, Minneapolis, MN, USA). The data were presented as mean value ± standard deviation.

## 4. Conclusions

The optimization of the acid treatment of dried brown seaweed biomass regarding the alginate yield was successfully achieved using the BBD. The main contribution of this study was the development of an innovative and efficient ultrasound-based procedure for alginate isolation for a relatively shorter time compared to conventional procedures. The liquid-to-solid ratio had the greatest influence on the alginate yield, followed by treatment time and treatment temperature. Unlike the positive effect of treatment time on alginate yield, the liquid-to-solid ratio and treatment temperature had negative effects. Under the proposed optimal conditions, i.e., a treatment time of 30 min, a liquid-to-solid ratio of 30 mL/g, and a treatment temperature of 47 °C, the determined alginate yield was 30.9% which was in line with the predicted value of 31.3%. The possibility of making further use of the ethanolic fraction representing a byproduct during alginate isolation from brown seaweed was also examined. Analysis of its antioxidant potential showed that the antioxidant-enriched extract was almost similar to the synthetic antioxidant BHT. The extract was also rich in lipids, some of which were identified using the UHPLC-ESI-MS/MS method. Based on the obtained results, the ethanol fraction with this composition and these properties could be further used as a fertilizer for the growth of different plant species, as well as for the development of products intended to be antifouling agents, applicable in aquaculture. This approach supports the circular economy concept because the huge amount of waste produced during alginate isolation can be further applied without uncontrolled release into the environment.

## Figures and Tables

**Table 1:** The Box–Behnken design matrix with system response (alginate yield).

Run	Factor 1	Factor 2	Factor 3	Response (Y): Alginate Yield (%)
	A: Treatment Time (min)	B: Liquid-to-Solid Ratio (mL/g)	C: Treatment Temperature (°C)	Experimental
1	20	10	70	29.91 ± 0.23
2	30	10	55	20.39 ± 0.11
3 *	20	20	55	13.12 ± 0.09
4	30	20	70	16.28 ± 0.10
5	10	20	40	16.78 ± 0.05
6	30	20	40	18.23 ± 0.13
7 *	20	20	55	14.61 ± 0.04
8 *	20	20	55	15.80 ± 0.19
9	10	10	55	27.98 ± 0.24
10	30	30	55	27.63 ± 0.02
11	20	30	40	28.88 ± 0.15
12	20	30	70	9.91 ± 0.03
13 *	20	20	55	13.59 ± 0.10
14	10	20	70	10.92 ± 0.05
15	10	30	55	9.40 ± 0.06
16	20	10	40	19.17 ± 0.10
17 *	20	20	55	15.14 ± 0.13

*—the central point of the BBD.

**Table 2:** ANOVA test for the second-order polynomial model.

	Sum of Squares	df	Mean Squares	F-Value	*p*-Value
Model	716.2	9	79.6	83.3	<0.0001 *
A-A	37.6	1	37.6	39.4	0.0004 *
B-B	58.8	1	58.8	61.6	0.0001 *
C-C	32.7	1	32.7	34.2	0.0006 *
AB	165.4	1	165.4	173.2	<0.0001 *
AC	4.0	1	4.0	4.2	0.0795
BC	222.2	1	222.2	232.7	<0.0001 *
A^2^	0.3	1	0.3	0.3	0.6190
B^2^	187.2	1	187.2	196.0	<0.0001 *
C^2^	3.3	1	3.3	3.4	0.1073
Residual	6.7	7	1.0		
Lack-of-fit	1.9	3	0.6	0.5	0.677
Pure error	4.7	4	1.2		
Cor total	722.8	16			
Standard deviation	0.9772		R^2^		0.9908
Mean value	18.09		adjusted R^2^		0.9789
CV %	5.40		predicted R^2^		0.9468
			Adequate precision		27.1

A—treatment time; B—liquid-to-solid ratio; C—treatment temperature; df—degree of freedom; Cor total—corrected total sum of squares; *—statistically significant value (*p* < 0.05).

**Table 3:** Identified extracts’ chemical constituents of brown seaweed with molecular/adduct ions and their proper fragment ions obtained after the effect of 35 eV collision energy. Numbers in parentheses (C:N) indicate the number of carbon atoms (C) and double bonds (N) in the fatty acid side chains. t_r_ is the retention time.

No.	[M − H]^−^ or [M + HCOOH]^−^ *m*/*z*	t_R_, min	MS/MS Fragment Ions, *m*/*z*	Compound	The Literature
1.	180.90	0.86	163, 143, 131, 119, 101 (100%), 89, 83, 59	d-(−)-mannitol *	
2.	236.93	6.89	193 (100%)	7-hydroxyflavone *	
3.	260.95	7.20	189, 165 (100%)	4-hydroxybenzoic acid-4-*O*-glucoside	Chen et al. [28]
4.	265.12	14.94	97 (100%)	oxidized fatty acid *	
5.	272.92 [M − H + HCOOH]^−^	0.65	227, 217, 159 (100%), 115	resveratrol	de Oliveira et al. [29]
6.	277.01	1.56	185 (100%), 141, 97	octadecatrienoic acid	Fahmy et al. [30]
7.	293.04	13.59	249, 236 (100%), 221, 217, 193, 136	hydroxy-octadecatrienoic acid	Fahmy et al. [30]
8.	304.87	19.00	175 (100%), 131	2-isopropylmalic acid derivative	Sobeh et al. [31]
9.	305.04	11.00	249, 231 (100%)	unknown	
10.	311.22	15.48	243, 211, 183, 149 (100%)	caftaric acid	Ramabulana et al. [32]
11.	317.03	15.11	299, 255, 249 (100%), 228, 215, 181, 163, 135	*p*-coumaric acid derivative *	
12.	319.18	15.61	301 (100%), 275, 257, 203, 179, 167, 115	5-*O*-*p*-coumaroylshikimic acid	Ben Said et al. [33]
13.	351.03	14.14	315 (100%), 297, 222, 161, 111	isorhamnetin derivative	Ben Said et al. [33]
14.	353.21	14.20	335, 317, 310, 256, 191 (100%), 173, 123	1-caffeoylquinic acid	Zhu et al. [34]
15.	355.30	16.54	337, 309 (100%), 205, 130	coumaroylglucaric acid isomer	Chandradevan et al. [35]
16.	360.93	6.02	317 (100%), 293, 273, 231, 185	phlorotannin derivative	Chen et al. [36]
17.	372.83	18.91	305 (100%)	fucophlorethol	Chen et al. [36]
18.	378.69	18.89	311 (100%), 249, 179	caftaric acid derivative	Ramabulana et al. [32]
19.	379.12	0.70	343 (100%), 179	quercetin derivative	Ben Said et al. [33]
20.	384.77	17.84	317, 249 (100%)	phlorotannin derivative	Chen et al. [36]
21.	385.20	16.29	317, 249, 227 (100%), 195, 175, 157	phlorotannin derivative	Chen et al. [36]
22.	439.29	17.52	281 (100%), 227	sterol (C:N 25:0;*O*6)	Fahmy et al. [30]
23.	440.87	18.94	425, 373, 305 (100%), 175	epigallocatechin derivative	Chandradevan et al. [35]
24.	452.73	17.66	385, 317 (100%), 249	phlorotannin derivative	Fahmy et al. [30]
25.	484.95	6.38	441, 397, 349, 315, 301 (100%), 297, 271, 257, 225	quercetin derivative *	
26.	501.12	14.84	484, 458, 433, 380 (100%), 365, 285, 272, 212, 197	kaempferol derivative	Chandradevan et al. [35]
27.	508.70	18.70	441 (100%), 373, 312, 305	epigallocatechin derivative	Chandradevan et al. [35]
28.	520.65	17.69	452, 387 (100%), 317	kaempferol-3-*O*-glucoside	Chandradevan et al. [35]
29.	527.09	7.15	499, 481, 409, 401 (100%), 387, 367, 341, 329, 313, 282, 271	unknown	
30.	527.30	15.21	334, 299, 244, 225 (100%), 207, 165, 153	orientin-sulphate	Ben Said et al. [33]
31.	547.40	17.07	479, 379, 285 (100%), 279, 267	luteolin derivative	Chandradevan et al. [35]
32.	549.45	18.16	482, 405, 287, 279 (100%), 269	liquiritigenin-hexose-xyl/ara	Wang et al. [37]
33.	555.32	16.27	508, 299 (100%), 225	sulfoquinovosyl monoacylglycerol (C:N 16:0)	Fahmy et al. [30]
34.	557.14	15.46	525, 511 (100%), 421, 308, 287, 275, 253, 231, 161	caffeic acid derivative	Chandradevan et al. [35]
35.	559.15	15.75	513 (100%), 491, 423, 289, 277, 253	caffeic acid derivative	Chandradevan et al. [35]
36.	571.31	17.40	521, 449, 311, 293 (100%), 277, 231	caffeic acid derivative	Chandradevan et al. [35]
37.	579.25	15.90	307, 225 (100%)	sulfoquinovosyl monoacylglycerol (C:N 18:2)	Cerulli et al. [38]
38.	581.30	16.32	535, 377, 308, 299 (100%), 282, 225, 208	3-*O*-methylorobol derivative	Ben Said et al. [33]
39.	588.70	17.64	521 (100%), 453, 317	unknown	
40.	599.06	10.29	555, 534, 507, 473 (100%), 459, 439, 411, 399, 383, 355, 343, 312, 275, 254	unknown	
41.	609.02	6.60	565 (100%), 439, 395, 301, 297	Rutin	Gašić et al. [39]
42.	665.46	17.88	634, 619, 605, 561, 545, 510, 445, 354 (100%), 337, 310, 293, 282, 266, 251, 239, 228	posphatidic acid (C:N 34:4)	Melo et al. [40]
43.	719.09	15.18	673, 583, 447 (100%), 397, 243	posphatidylglycerol (C:N 32:1)	Melo et al. [40]
44.	721.15 [M + HCOO]^−^	15.50	675 (100%), 653, 397	digalactosyl monoacylglycerol (C:N 18:3)	Fahmy et al. [30]
45.	723.10	15.92	677, 452	phlorotannin sulfate	Chouh et al. [41]
46.	725.14	16.52	689, 679, 589, 453, 429 (100%), 397, 379	unknown	
47.	774.00	17.02	737 (100%), 688, 455, 443	unknown	
48.	805.43	16.94	761, 391 (100%), 347, 305	unknown	

* https://massbank.eu (accessed on 9 May 2024).

**Table 4:** The concentrations of different elements within the examined formulations.

Macroelements	Macroelement Content (mg/g d.w.)	Microelements	Microelement Content (μg/g d.w.)
Ca	0.11 ± 0.006	Al	1.92 ± 0.19
K	36.77 ± 0.92	B	198.84 ± 2.51
Mg	11.90 ± 0.43	Ba	0.07 ± 0.01
Na	106.59 ± 1.23	Cd	0.05 ± 0.004
P	4.57 ± 0.16	Co	0.03 ± 0.003
S	18.88 ± 0.25	Cr	0.09 ± 0.005
Si	0.03 ± 0.002	Cu	1.50 ± 0.01
		Fe	17.26 ± 0.48
		Li	1.24 ± 0.02
		Mn	8.80 ± 0.11
		Ni	0.62 ± 0.05
		Pb	0.48 ± 0.03
		Sr	1.49 ± 0.13
		Zn	7.06 ± 0.21
Total macroelements	178.84	Total microelements	239.45

**Table 5:** Minimum inhibitory concentration (MIC), minimum bactericidal concentration (MBC), and minimum fungicidal concentration (MFC) values (in mg/mL) of ethanolic fraction of brown seaweed biomass after extraction of alginate.

Microorganism	Sample		Gentamicin	
	MIC (mg/mL)	MBC (mg/mL)	MIC (mg/mL)	MBC (mg/mL)
*Staphylococcus aureus* ATCC 6538	5	10	0.125	/
*Enterococcus faecalis* ATCC 29219	10	20	0.062	0.062
*Bacillus cereus* ATCC 10876	5	10	0.015	0.125
*Escherichia coli* ATCC 25922	10	10	0.015	0.015
*Pseudomonas aeruginosa* ATCC 27853	10	20	0.500	1.000
*Shigella sonnei* ATCC 29930	10	20	0.078	0.031
			Nystatin	
	MIC (mg/mL)	MFC (mg/mL)	MIC (mg/mL)	MFC (mg/mL)
*Candida albicans* ATCC 1231	20	/	0.0077	0.0625

**Table 6:** Factor levels of independent variables.

Factors	Coded Values		
	−1	0	+1
	Actual Values		
Treatment time [min]	10	20	30
Liquid-to-solid ratio [mL/g]	10	20	30
Treatment temperature [°C]	40	55	70

## Data Availability

The original contributions presented in the study are included in the article, further inquiries can be directed to the corresponding authors.
